# Matrix Metalloproteinases in Non-Neoplastic Disorders

**DOI:** 10.3390/ijms17071178

**Published:** 2016-07-21

**Authors:** Akinori Tokito, Michihisa Jougasaki

**Affiliations:** Institute for Clinical Research, National Hospital Organization Kagoshima Medical Center, 8-1 Shiroyama-cho, Kagoshima 892-0853, Japan; kenkyubu@kagomc2.hosp.go.jp

**Keywords:** matrix metalloproteinase, non-neoplastic disease, therapeutic potential

## Abstract

The matrix metalloproteinases (MMPs) are zinc-dependent endopeptidases belonging to the metzincin superfamily. There are at least 23 members of MMPs ever reported in human, and they and their substrates are widely expressed in many tissues. Recent growing evidence has established that MMP not only can degrade a variety of components of extracellular matrix, but also can cleave and activate various non-matrix proteins, including cytokines, chemokines and growth factors, contributing to both physiological and pathological processes. In normal conditions, MMP expression and activity are tightly regulated via interactions between their activators and inhibitors. Imbalance among these factors, however, results in dysregulated MMP activity, which causes tissue destruction and functional alteration or local inflammation, leading to the development of diverse diseases, such as cardiovascular disease, arthritis, neurodegenerative disease, as well as cancer. This article focuses on the accumulated evidence supporting a wide range of roles of MMPs in various non-neoplastic diseases and provides an outlook on the therapeutic potential of inhibiting MMP action.

## 1. Introduction

Matrix metalloproteinases (MMPs, also known as matrixins) are secreted or membrane-bound endopeptidases belonging to the metzincin superfamily. Other members of the superfamily are adamlysins, including a proteinase with a disintegrin and metalloproteinases (ADAMs), ADAM with thrombospondin-like motifs (ADAMTSs), astacins, serralysins and pappalysins. As their names indicate, these enzymes contain zinc ions in their structure, which are needed to carry out the hydrolysis of protein substrates. Currently, MMPs consist of 23 members in human and are expressed in almost all organs and tissues [1].

The first MMP was reported by Gross and Lapiere in 1962 as a collagenolytic agent engaged in tail resorption during the tadpole metamorphosis [2]. Since then, MMPs have been widely known for their ability to cleave a wide range of extracellular matrix (ECM) components and thereby contributing to tissue turnover. However, in the past few years, a growing number of non-matrix substrates for MMPs, including cytokines/chemokines, growth factors, antimicrobial peptides, signaling receptors and other membrane proteins, have been uncovered. MMPs can activate or mediate the functions of these proteins not solely as proteinases, but rather as extracellular processing enzymes. Because of the diversity of their substrates and the widespread distribution of these proteinases, MMPs are involved in various homeostatic functions, for example tissue remodeling, wound healing and immunity. Therefore, it becomes increasingly clear that the role of MMPs in vivo is more significant and complicated.

**Figure 1:**
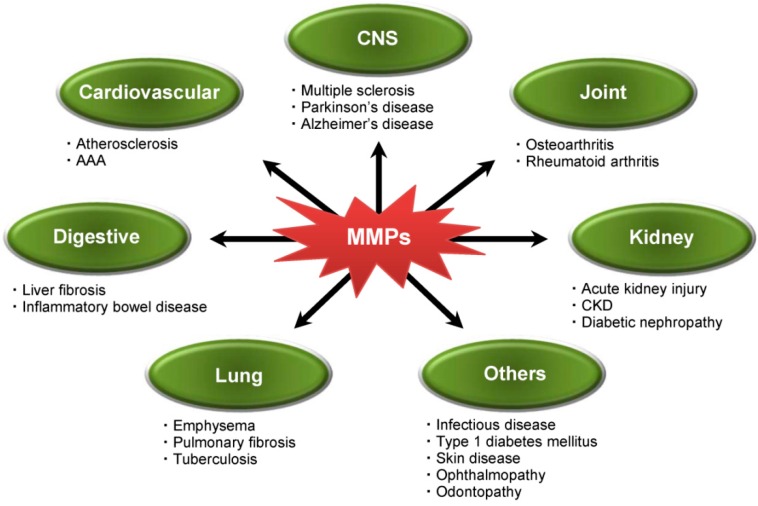
Involvement of matrix metalloproteinases (MMPs) in non-neoplastic disorders. AAA, abdominal aortic aneurysm; CKD, chronic kidney disease.

The expression and activities of MMPs are strictly controlled under normal circumstances. On the other hand, their abnormal activation or over-production are detrimental and often noted in many pathological processes involved in systemic non-neoplastic disorders, such as cardiovascular diseases, arthritis and neurodegenerative diseases, as well as cancer (Figure 1). Hence, MMPs have been considered as an attractive therapeutic target for the treatment of these diseases.

The aim of this review is to summarize the accumulated knowledge about the MMPs, when necessary added with ADAMs and ADAMTSs, discussing their structures, functions, regulation and pathological roles in the common non-neoplastic disorders. Since the over-expression and detrimental roles of MMPs in cancer have been extensively reviewed [3,4,5], the issue is beyond the scope of this paper.

## 2. Matrix Metalloproteinases

### 2.1. Structure and Classification

**Figure 2:**
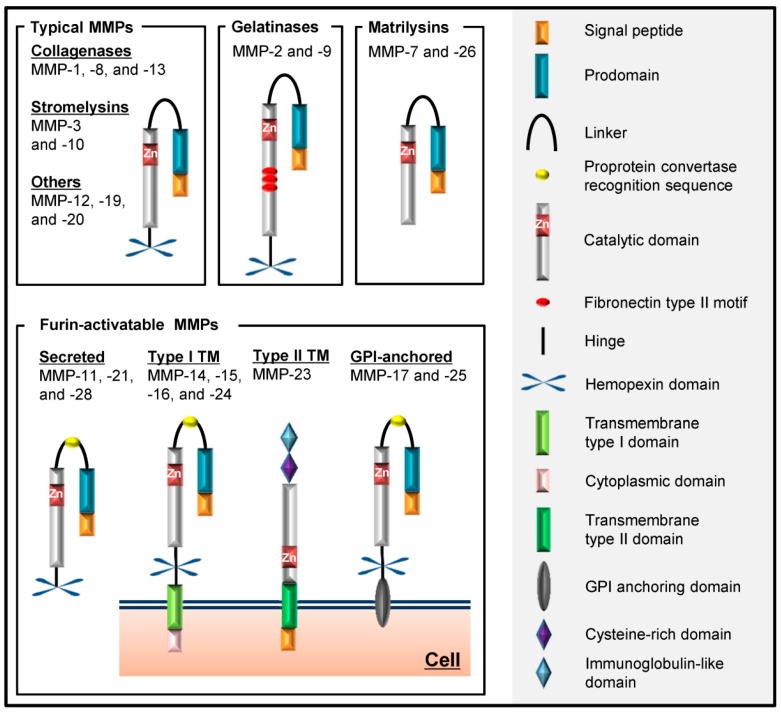
The schematic structures of the MMP family. Matrix metalloproteinases (MMPs) have four basic domains: the signal peptide responsible for secretion, the prodomain, which keeps the MMP inactive by coordinating the zinc ion of the catalytic site, the catalytic domain responsible for the proteolytic activity and the hemopexin domain of a propeller blade structure. The gelatinases contain three fibronectin type II repeats, which bind gelatin. Matrilysins and MMP-23 lack the hinge region and the hemopexin domain. The membrane-type MMPs (MT-MMPs) are localized on the cell surface anchored by a transmembrane (TM) domain or a glycosylphosphatidyl-inositol (GPI) anchor.

Until now, 23 distinct proteinases have been identified as the members of the MMP family in human [1]. The MMP members can be classified in different fashions. The most common classification is based on the substrate specificity and the cellular localization of each MMP, such as collagenases, gelatinases, stromelysins and membrane-type MMPs (MT-MMPs). However, several MMPs cannot fit into any of these traditional groups. On the other hand, MMPs can also be classified on the basis of their structures as typical MMPs, gelatinases, matrilysins or furin-activatable MMPs. The typical MMPs can be further divided into three subgroups on the basis of their substrate specificity as collagenases, stromelysins and others (Figure 2). In particular, MMP-1, -8 and -13 are categorized as collagenases; MMP-2 and -9 are gelatinases (gelatinase A and B, respectively); MMP-3 and -10 are stromelysins (stromelysin-1 and -2, respectively); MMP-7 and -26 are matrilysins (matrilysin-1 and -2, respectively). Among furin-activatable MMPs, except for MMP-11, -21 and -28, which are secreted after intracellularly activation, MMP-14, -15, -16 and -24 are membrane-type MMPs with a type I transmembrane domain (MT1-, MT2-, MT3- and MT5-MMP, respectively); MMP-23 is another membrane-type MMP with a type II transmembrane domain; and MMP-17 and -25 are glycosylphosphatidyl-inositol (GPI)-anchored MMPs (MT4- and MT6-MMP, respectively). MMP-12, -19, -20 and -27 are the other MMPs [6].

MMPs are highly homologous in their structure. Most of them have the four basic domains: signal peptide, autoinhibitory prodomain, catalytic domain and hemopexin-like domain. The N-terminal signal peptide is needed to traffic the nascent MMP to the endoplasmic reticulum and transport it out of the cell. All MMPs have the catalytic domain responsible for the proteolytic activity, which is shielded in the latent form by the prodomain. This prodomain contains a thiol of the conserved cysteine residue that coordinates the zinc ion of the catalytic site [7]. This interaction, named the “cysteine switch”, thus prevents the latent form from becoming the active form. When the prodomain is destabilized or removed, MMPs become functionally active. MMP-26 is the only MMP that does not have this cysteine switch mechanism to keep the enzyme in the latent form [8]. MMPs, except matrilysins and MMP-23, have a flexible proline-rich hinge region and a C-terminal hemopexin-like domain, which functions in substrate recognition [9]. Otherwise, the gelatinases contain a series of three fibronectin type II repeats inserted in the catalytic domain, which allow the ability to bind collagen and denatured collagen (gelatin) [10]. Although most MMPs are secreted molecules, MT-MMPs are localized on the cell surface anchored by a single-pass transmembrane domain followed by a short cytoplasmic tail or by a GPI linkage [11]. MMP-23 has unique features, such as an amino-terminal signal anchor, which targets MMP-23 to the cell membrane, a cysteine-rich region and an immunoglobulin-like domain in place of the hemopexin-like domain.

### 2.2. Substrate Specificity and Function

MMPs target a wide range of ECM components, thereby playing a critical role in tissue remodeling. In particular, collagenases are able to cleave all fibrillar collagens (types I, II and III) at their triple-helical domain, though there are slight differences in their specific activities to each collagen molecule. The preferred substrate for MMP-13, for example, is collagen II [12]. Gelatinases can degrade gelatin, which is identical to denatured collagen. Stromelysins can digest a wide array of substrates, such as gelatin, fibronectin, nidogen, laminin, tenascin, vitronectin and decorin, as well as proteoglycan [13]. Among matrilysins, MMP-7 targets proteoglycan fibronectin, type IV collagen, laminin and entactin. Another matrilysin, MMP-26, has more restricted substrate specificity compared to MMP-7. Among MT-MMP members, MMP-14 and -15 have largely overlapping substrate specificity, including native fibrillar collagen, gelatin, fibronectin and vitronectin. MMP-17 and -25 linking to the cell membrane by a GPI anchor cleave type IV collagen, gelatin, fibronectin and laminin [14]. It is to be noted that these functions are based on the in vitro data and, therefore, do not always predict in vivo functions.

For the specific binding of substrates with certain MMP, MMPs possess several subsites (S), named as unprimed S1, S2 and S3 (left-hand side of the Zn^2+^ ion) and primed S1′, S2′ and S3′ (right-hand side of the Zn^2+^ ion), as well as the Zn^2+^ ion in their catalytic site [6]. Among them, the S1′ pocket is the most important recognition site, at least for small synthetic substrates, which is characterized by its variation according to the different MMPs in both the amino acid sequence and the depth of the pocket (shallow, intermediate and deep pocket) [15]. For all MMPs, the key determinant of the cleavage position of peptide substrates is the P1′–S1′ interaction (P1′ is the group in the substrate binding to the S1′ pocket of the enzyme) [16].

MMPs are extremely pleiotropic enzymes. Their proteolytic activity against matrix and non-matrix substrates mentioned above contributes to the regulation of tissue architecture or creates a space for cells to migrate via effects on the ECMs and intercellular junctions. In addition, it can mediate the bioactive state and local delivery of signaling molecules, either directly or indirectly [11]. Further, ectodomain shedding of cell surface receptors by MMPs affects the surface composition of the plasma membrane and the responsiveness of a cell to extracellular signals [1]. Interestingly, their proteolytic action also generates resultant cleavage fragments with independent biological activity [17,18]. Given these multiple effects, it seems plausible that MMPs can play an important role in diverse physiological and pathophysiological conditions.

### 2.3. Regulation of MMP Expression and Activity

MMPs are produced by various types of cells, which include inflammatory, stromal and epithelial/endothelial cells. Some members, such as MMP-2, -19, -28 and several MT-MMPs, are detectable in normal tissues, implying their roles in homeostasis. However, most MMPs are induced in response to tissue injury or infection and function in a wide range of wound repair, inflammation and defense processes against the external environment [19].

The catalytic activity of MMPs is controlled at several levels involving gene expression, zymogen activation, compartmentalization and inhibition of active enzyme [19]. The production of MMPs is initially and predominantly regulated at the transcriptional level by a variety of physiological triggers, including growth factors, cytokines, chemokines, hormones, tumor promoters and cell–cell or cell–ECM interactions [20]. MMP promoters contain cis-acting elements that can bind several transcription factors, such as activator protein 1 and nuclear factor (NF)-κB [21]. Expression of several MMPs is further regulated at the post-transcriptional level by modulating mRNA stability [22]. Recently, the contribution of epigenetic modifications of MMP has also been uncovered [23].

Generally, MMPs are synthesized and secreted as inactive zymogens and are subsequently activated through several mechanisms, causing disruption of their cysteine switch, i.e., the covalent bond between the thiol of the conserved cysteine residue in the prodomain and the zinc ion in the catalytic site. The cysteine switch can be activated by cleavage of the prodomain by itself (autolysis) or by other proteinases, such as plasmin, trypsin, furin and other MMPs. In addition, the cysteine switch can be broken by the oxidation of cysteine by reactive oxygen species or artificially broken by mercury-containing compounds, leading to allosteric relocation of the prodomain, resulting in the autolytic cleavage [24]. MMP activity and specificity are also controlled through the compartmentalization in a certain intracellular or extracellular location, possibly via their interaction with glycosaminoglycans [25].

After activation, MMPs can be inhibited by both general protease inhibitors, such as α_2_-macroglobulin and their specific inhibitors, i.e., tissue inhibitors of metalloproteinases (TIMPs). The TIMPs are a family consisting of four members (TIMP-1, -2, -3 and -4), which bind to MMPs in a 1:1 stoichiometric ratio and competitively and reversibly inhibit the activity of all MMPs [26]. TIMPs exhibit a tissue-specific distribution reflecting their gene expression, which is individually regulated. TIMP-3 is sequestered to ECMs, whereas all other TIMPs are present in soluble form. There is considerable overlap in the biochemical property of TIMPs, although there are some specificities, for example the specificity of TIMP-3 for ADAM17 [1]. Besides their inhibitory activity, TIMPs have other important biological functions involved in cell proliferation and apoptosis, angiogenesis and even activation of several latent proMMPs [27]. Ultimately, the MMP/TIMP balance is an important factor in controlling the overall proteolytic activity in vivo.

## 3. ADAMs and ADAMTSs

The ADAMs and ADAMTSs are distinct proteinase families, both of which belong to the metzincin superfamily. Until now, 21 *ADAM* and 24 *ADAMTS* protein-coding genes have been identified in human genome [28]. As their names indicate, ADAMs and ADAMTSs are structurally similar and share several domains, including prodomain, zinc-peptidase domain and disintegrin domain, through which they play multiple biological roles in cells. Despite the presence of metallopeptidase domains, only 13 of 21 ADAMs exhibit proteolytic activity. On the other hand, five of 24 ADAMTS lack the metallopeptidase domains and, therefore, are proteolytically inactive.

ADAMs are membrane-anchored proteins involved in activating zymogens, such as tumor necrosis factor (TNF)-α, epidermal growth factor and amyloid precursor protein (APP), by shedding of their ectodomains [29]. ADAMs also participate in cell adhesion and fusion via interaction with integrins in neighboring cells. In contrast, ADAMTSs are secreted proteins mainly responsible for ECM maintenance by degrading specific matrix components such as procollagen, hyalectan and proteoglycan. Because seven ADAMTSs, including ADAMTS-1, -4, -5, -8, -9, -15 and -20, can cleave large aggregating proteoglycans (known as aggrecans), these enzymes are regarded as aggrecanases [30]. Indeed, ADAMTS-4 and ADAMTS-5 are called aggrecanase-1 and aggrecanase-2, respectively. Furthermore, ADAMTSs are also implicated in the coagulation system by cleaving von Willebrand factor precursor protein [31]. Like MMPs, ADAMs and ADAMTSs proteinases have gradually been recognized to be involved in a number of pathophysiological processes mentioned below in this context.

## 4. Involvement of MMPs, ADAMs and ADAMTSs in Non-Neoplastic Diseases

### 4.1. Cardiovascular Diseases

#### 4.1.1. Atherosclerosis

Atherosclerotic disease, such as acute coronary syndrome and stroke, is the leading cause of morbidity and mortality in adults. Atherosclerosis is widely recognized as an inflammatory process occurring in several distinct steps [32], many of which are associated with alterations in MMP activity [33]. All cells present in the normal and pathological blood vessel wall upregulate and activate MMPs in a multistep fashion driven in part by inflammatory mediators, including angiotensin II and cell–cell interactions [34]. These activated MMPs can degrade the vascular ECM components, such as collagen, elastin, probably resulting in aging, hypertension and atherogenesis within the arterial wall [35]. In the initial stages of this disease, MMP activation contributes to intimal growth and vessel wall remodeling in response to injury, most notably by promoting the migration of vascular smooth muscle cells (VSMCs). Further, a broader spectrum and higher level of MMP activation associated with inflammation could cause plaque rupture in later phases of atherosclerosis. The rupture of a plaque, a trigger for the onset of cardiovascular disorders, is largely based on the instability of the plaque, which is rich in lipids, and on the structural vulnerability caused by alteration in the balance between accumulation and degradation of ECM.

##### Formation of the Atherosclerotic Plaque

After disruption of the vascular barrier of endothelial cells due to injury, various inflammatory cells and plasma proteins, including lipoproteins, translocate across the vessel wall into the surrounding tissues. In particular, monocytes penetrate the intima and turn into macrophages, which ingest lipids and eventually become foam cells, a major source of MMPs [36]. Secreted MMPs allow VSMC migration through the internal elastic lamina into the intima and also promote VSMC proliferation, thereby contributing to the growth of the atheroma. A large body of evidence has demonstrated an early upregulation of MMP-9 and activation of MMP-2 during neointima formation after vascular injury, and they are implicated in VSMCs migration and proliferation [37]. MMP-14 was also demonstrated to participate in vascular remodeling by promoting the migration and proliferation of arterial VSMCs [38,39]. Deficiency of MMP-2, -9 and -14 decreased intima hyperplasia in mouse models [40,41,42,43,44]. The roles of MMP-9 and -12 in promoting intima thickening by cleavage of *N*-cadherin leading to SMC proliferation have also been demonstrated [45]. Recently, Johnson et al. showed that MMP-3-mediated activation of MMP-9 was necessary for efficient neointima formation [46]. Furthermore, MMP-8 deficiency in ApoE null mice resulted in smaller lesions, and *MMP-8* gene variation in patients with coronary artery disease was associated with the extent of coronary atherosclerosis [47]. On the other hand, transgenic overexpression of MMP-1 in macrophages reduced the progression of atherosclerosis in ApoE knockout mice by diminishing the content of fibrillar collagen [48]. In addition, MMP-3 deficiency enlarged atherosclerotic plaque size in the same mice model [49]. These findings suggest that both activities of MMP-1 and -3 may have protective effects against plaque formation by the degradation of matrix components.

Studies investigating the role of ADAM and ADAMTS in atherosclerotic plaque formation are still limited. However, upregulation of ADAM-15 and ADAM-9 in human atheroma has been observed [50], and increased ADAM-17 expression was associated with atherosclerosis resistance in LDL-receptor-deficient mice [51]. It has also been shown that ADAMTS-1 and ADAMTS-7 facilitate VSMC migration and neointima formation in human and rat [52].

##### Atherosclerotic Plaque Destabilization

The fibrous cap overlying an atheromatous core of lipid consists of the ECM components, such as collagen and elastin, which serve the purpose of strength and flexibility. Under stable conditions, a remodeling of the fibrous cap is well balanced between the matrix degradative and constructive processes, and thus, the plaque protects the lumen of the vessel from the exposure of the underlying thrombogenic materials. However, under proinflammatory conditions, the balance can be altered in favor of the degradation, leading to a dangerous weakness in the fibrous cap and eventually resulting in plaque rupture [53].

Several MMPs play a central role in plaque rupture. Among them, MMP-2 and MMP-9 are largely known as causes of plaque destabilization. Analysis of human coronary atherectomy specimens revealed increased expression of MMP-9 in the plaques of patients with acute coronary syndromes, but not in those of patients with stable angina [54]. Serum levels of MMP-2 and -9 were elevated in carotid endarterectomy patients with neurologic symptoms compared to those without symptoms, and especially, MMP-9 was highly associated with histological markers of unstable plaque structure [55]. More recently, it was also demonstrated that F-fluorodeoxyglucose positron emission tomography could predict high-risk lipid-rich and hemorrhagic plaques, which are associated with strong immunoreactivity against MMP-9, as well as CD68 [56]. Furthermore, overexpression of an autoactivating form of MMP-9 in macrophages induced plaque disruption in ApoE−/− mice [57]. Several drugs, such as angiotensin type II receptor blocker and statin, whose protective efficacy against cardiovascular diseases is widely known, have been reported to reduce MMP-2 and -9 activities and, thereby, contribute to the protection against plaque rupture [58,59].

On the other hand, ApoE null mice overexpressing human MMP-1 in macrophages showed smaller, but destabilized plaques with fewer cellular layers and diminished content of fibrillar collagen compared to control littermates [48]. Moreover, MMP-7, -8, -12, -13, -14, as well as MMP-9 increase matrix degradation, inflammation and/or apoptosis, leading to plaque rupture [60]. Consistent with these results, overexpression of human TIMP-1 or TIMP-2 has been reported to inhibit plaque destabilization [61,62]. Interestingly, studies with specific MMP knockout animals in the ApoE null background have documented that some MMP has either a protective or a deleterious effect in plaque stability, and some have both effects [60].

Causative factors responsible for the localized enhancement of MMP expression and activity in unstable plaques seem to be oxidative stress, thrombin and plasmin formation. For example, thrombin production resulting from plaque rupture leads to MMP-2 activation, and subsequently, this enzyme may in turn increase platelet activation, resulting in further generation of thrombin and secondary activation of MMP-2 [63].

#### 4.1.2. Abdominal Aortic Aneurysm

Abdominal aortic aneurysm (AAA) represents another major cause of cardiovascular-related morbidity and mortality. The underlying pathogenesis of AAA is complex, but a key contributor to its formation is protease-driven destruction of elastin fibers and interstitial collagens (type I and III) in the media and adventitia, leading to a weakening of the vessel wall, resulting in the progressive expansion and rupture of AAA [64].

In human, the expression of multiple MMPs, including MMP-1, -2, -3, -9, -12, -13 and -14, is increased in aneurysmal tissues [65,66,67,68,69,70]. An in vivo molecular imaging of MMP activity within the walls of AAA in mice showed a linear relationship between proteolytic activity and aneurysmal growth, suggesting that MMP plays a pivotal role in the pathophysiology of the disease [71]. In addition, studies using specific MMP knockout mice showed that especially MMP-2 and -9 were highly implicated in the development and progression of AAA [72,73]. MMP-2 and -9 are able to erode the arterial wall, a histological foundation of aortic aneurysm development, and promote a loss of vascular SMCs, leading to aortic dilatation [35]. MMP-9 was also reported to be associated with the susceptibility to AAA rupture, but not MMP-2 [74]. Most recently, Mata et al. showed that the animals treated with doxycycline, a nonselective MMP inhibitor, showed an 85% decrease in AAA development, which was associated with a large reduction in gelatinolytic activity of MMP-2 and -9 and decreased inflammatory response [75]. On the other hand, TIMP-1, the primary endogenous inhibitor of MMP-9, has a protective effect on AAA formation, suggested by the finding that TIMP-1-deficient mice developed larger aneurysms after elastase infusion compared to wild-type mice [76]. Intriguingly, protease-driven destruction of elastin fibers results in a release of elastin degradation product, which is a biologically-active matrikine having a vital role in the chemotaxis of inflammatory cells, the release of MMP-1 and -2 and SMC proliferation. These processes may additionally contribute to the development of AAA [77].

### 4.2. Joint Disorders

The joint disorders, such as osteoarthritis (OA) and rheumatoid arthritis (RA), are common causes of physiological disability accompanied by pain, leading to impaired quality of life. In both OA and RA, excessive degradation and loss of cartilage ECM are known to be the pathological hallmark, which ultimately result in the progressive remodeling of affected joints [78].

Cartilage ECM is synthesized by the chondrocytes and mainly composed of type II collagen and aggrecan. Type II collagen is associated with the tensile strength of cartilage. Aggrecan is the most abundant proteoglycan in articular cartilage and provides cartilage with a load-bearing property. The cartilage ECM also constitutes a signaling scaffold, which is essential for the maintenance of the phenotypic stability of chondrocytes and their metabolic activity [79]. Although a number of proteinases are produced in the arthritic joint, specific MMPs and ADAMTSs have been suggested to play a central role in the degradation of cartilage ECM components. In fact, collagenolytic MMPs (MMP-1, -2, -8, -13 and -14) can degrade type II collagen, whereas aggrecan can be degraded by aggrecanase-type ADAMTSs (ADAMTS-1, -4, -5, -8, -9, -15, -16 and -18), as well as MMPs (MMP-1, -2, -3, -7, -8, -9 and -13) [80]. MMP-2, a major genatinase, can also cleave fibrillar collagen, and its contribution to joint destruction has been reported [81,82]. The aggrecan degradation can be reversed, but the collagen degradation is irreversible, because articular cartilage cannot be repaired once collagen is lost [83].

#### 4.2.1. Osteoarthritis

OA is a degenerative joint disease found in the elderly, which is triggered by accumulated mechanical stresses to joints, leading to the destruction of the articular cartilage by proteolytic enzymes secreted from activated chondrocytes embedded in itself [84]. Of the two components of cartilage ECM, aggrecan is degraded in the initial phase of OA, whereas type II collagen is degraded in the later phase [80].

Among aggrecanase-type ADAMTSs, ADAMTS-4 and -5 are major players in development of OA. Double knockout of ADAMTS-4 and -5 in mice prevented the progression of OA, and this effect was comparable to that of single knockout mice of ADAMTS-5 [85], suggesting that ADAMTS-5 might be the primary aggrecanase in mice. In human OA cartilage, on the other hand, ADAMTS-4 is mainly overexpressed [86] and contributes to the degradation of cartilage aggrecan, as well as ADAMTS-5 [87]. Furthermore, the expression of ADAMTS-4 in human OA, but not ADAMTS-5, was induced by IL-1α and TNF-α, which are major cytokines involved in OA [88].

Among MMPs upregulated in human OA, MMP-3 and -13 have been the most strongly implicated MMPs in cartilage destruction because of their collagenolytic activity [78,80]. Elevated in vivo activity of MMP-13 in OA was recently confirmed by using an imaging probe of quenched fluorescent peptide substrate, and the activity correlated with the histological severity of this disease [89]. Transgenic overexpression of active MMP-13 in mouse articular cartilage resulted in OA-like pathological changes, showing increased cleavage of type II collagen [90]. The functional importance of MMP-13 in OA was also underlined by another study showing improved OA cartilage erosions in MMP-13-deficient mice [91]. On the other hand, MMP-3 might contribute to the pathological condition of OA indirectly by activating the latent forms of collagenases, such as MMP-1 and MMP-13, or directly by digesting many ECM components, including aggrecan and collagen [19].

#### 4.2.2. Rheumatoid Arthritis

RA is a systemic inflammatory disease afflicting numerous joints for reasons that are not fully understood. Systemic and local autoimmune reactions result in the activation of synovial cells, such as fibroblast-like synoviocytes (FLS) and monocytes, which attack the cartilage matrix [92,93]. The mediators of cartilage destruction in RA seem to overlap with those in OA, including MMPs, ADAMTSs and cathepsins, and their production mainly from synovial cells is greatly influenced by inflammatory mediators, cytokines and growth factors. MMP-14, a membrane-bound MMP (also called MT1-MMP), is highly expressed in FLS and macrophages from patients with RA [94] and appears to be a predominant MMP to degrade collagenous cartilage matrix [95]. In addition, the functional importance of MMP-14 has been demonstrated by studies using antisense and dominant-negative constructs for this enzyme showing the inhibition of synovial fibroblast invasion into RA cartilage [96,97]. Other matrix enzymes, in particular ADAMTS-4 and -5, are responsible for the degradation of aggrecan in RA [98] and, thus, further diminish the cartilage integrity [99].

Because of the crucial roles of MMPs in joint destruction, they have been regarded as useful biomarkers. Notably, serum levels of MMP-1 and -3 in RA patients have been well known to correlate with the disease activity [100], and thus, MMP-3 is a practically useful marker for predicting bone and cartilage damage and evaluating therapeutic efficacy in RA [101].

### 4.3. Neurodegenerative Diseases

The ECM in central nervous system (CNS) is composed mainly of proteoglycans and essential for neuronal cell development, survival and activity. MMPs are involved not only in remodeling and excessive degradation of brain ECM, but also in various biological and pathological processes in CNS, such as microglial activation, inflammation and blood-brain barrier (BBB) disruption [102,103,104]. Many forms of MMPs have been demonstrated to be widely expressed in the CNS [105]. Their expression levels in the normal adult brain are quite low, while upregulated in injury and in several neurological disorders, such as multiple sclerosis (MS), Alzheimer’s disease (AD) and Parkinson’s disease (PD), suggesting that MMPs play critical roles in their pathophysiological mechanisms [105,106,107,108]. Among these MMPs, MMP-2 and -9 have been mostly investigated on their effects in CNS, probably due to their easy detectability [109].

#### 4.3.1. Multiple Sclerosis

MS is an autoimmune disease characterized by demyelination and loss of axons, which causes non-traumatic disability in young adults [110]. Studies in both human with MS and animals of experimental autoimmune encephalomyelitis (EXE), a mimic disease of MS, have shown increased expression of many MMPs, both in vivo and in vitro [111,112,113,114,115,116]. MMPs play important roles in the pathophysiology of MS by disrupting BBB, leading to chronic inflammatory cascade, and by breaking down myelin sheath within CNS parenchyma [117]. For example, MMP-2 injection into rat brain caused the disruption of BBB and tissue destruction, and the effects was blocked by TIMP-2 [118]. In addition, MMP-2 and -9 double knockout mice, but neither MMP-2 nor -9 alone, failed to develop EXE [119,120]. MMP-8 deficiency or inhibition also showed a significant reduction of demyelinating lesion and inflammatory cell infiltration in the EXE mice [121]. In human studies, it is well established that MMP-9 is upregulated in cerebrospinal fluid (CSF), blood and brain tissue of MS patients compared to healthy controls [116,122,123,124,125]. Serum level of MMP-9 and the MMP-9/TIMP-1 ratio were proven to be useful biomarkers in various courses of MS [123], and they were lowered by treatment with interferon-β, a drug commonly used for MS [108]. Moreover, other MMPs, including MMP-1, -2, -3, -7 and -14, have also been reported to be upregulated in patients with MS and implicated in its pathology [108,116].

#### 4.3.2. Parkinson’s Disease

PD is one of the neurodegenerative disorder caused by selective loss of dopaminergic neurons in substantia nigra pars compacta and is clinically characterized by motor symptoms, such as resting tremor, unbending nature, bradykinesia and postural unsteadiness.

MMP-3 is well known as a possible contributor to PD pathophysiology via multiple mechanisms, including α-synuclein processing, microglial activation and BBB disruption [126]. α-Synuclein cleaved by MMP-3 can readily form the aggregate, which is the major component of the Lewy body of PD and is more cytotoxic than the uncleaved α-synuclein [127]. Both microglia activation and BBB disruption are triggers for neuroinflammation, consequently leading to the neurodegeneration in PD [128,129]. In addition, increased expression of MMP-9, as well as MMP-3 has been observed in various animal models of PD [127,130] and the C (-1562) T polymorphism in the *MMP-9* gene leading to higher promoter activity was associated with the risk for PD [131]. Furthermore, a recent study comparing MMP-12-deficient mouse with the wild-type demonstrated that upregulated cerebral MMP-12 during aging can likewise exacerbate neuroinflammation [132].

#### 4.3.3. Alzheimer’s Disease

AD is a major neurodegenerative cause of dementia. One of its pathological hallmarks is the deposition of amyloid-β peptide (Aβ) in extracellular senile plaques and vessels [133], and Aβ can induce several MMPs, such as MMP-2, -3 and -9, in blood vessels, astrocytes and microglia [134,135]. In fact, altered levels of MMPs have been reported in the plasma, CSF and brain tissue of AD patients compared to healthy controls [136,137]. Furthermore, growing evidence indicates that MMPs play an important role in both the formation and clearance of Aβ, thereby contributing to the pathophysiology of AD [138]. MMP-2 is a major MMP directly linked to the physiological catabolism of Aβ in brain. Its resulting proteolytic fragments are soluble and do not exhibit a fibrillogenic or a cytotoxic property in human cerebral microvascular endothelial or neuronal cells [139], suggesting that MMP-2 might have a protective role in AD. MMP-9 has also been indicated to exert a neuroprotective effect via Aβ clearance, reducing the aggregation of the peptide [140,141]. In contrast, however, Mizoguchi et al. demonstrated that Aβ-induced cognitive impairment was alleviated in MMP-9 knockout mice [142]. Moreover, in a human study, there were inverse correlations between the Global Cognitive Score or the Mini-Mental State Examination (MMSE) score and MMP-9 activity [143]. These data indicate another effect of MMP-9 as a neurotoxic molecule in AD. On the other hand, the Rotterdam Study showed that MMP-3 haplotypes were associated with alterations in plasma Aβ levels in human [144]. Because MMP-3 can activate proMMP-9, elevated brain levels of MMP-3 might result in enhancement of MMP-9 activity and indirectly affect AD progression. Additionally, it has also been indicated that MMP-14, -16 and -18 might play important roles in the regulation of APP function in CNS [145].

### 4.4. Digestive Disorders

#### 4.4.1. Liver Fibrosis

Liver fibrosis occurs as a result of the4 wound-healing response to chronic injury, including viral hepatitis and alcoholic/non-alcoholic steatohepatitis [146]. Following the injury, hepatic stellate cells (HSCs) become activated and produce a combination of MMPs and TIMPs, as well as a large amount of ECMs, especially fibrillar collagen type I and type III. Although HSCs initially exhibit a matrix-degrading phenotype, the pattern changes in chronic phases of liver injury, and the HSCs exhibit a pro-fibrotic phenotype by degrading normal liver matrix, while inhibiting the degradation of fibrillar collagens that accumulate in liver fibrosis [147].

As expected based on their proteolytic activity, various MMPs have been reported as anti-fibrotic enzymes in liver fibrosis. For example, overexpression of collagenases (MMP-1, -8 and -13) in a rat model of liver fibrosis was associated with the recovery from fibrosis and induced normal hepatocyte proliferation [148,149,150]. Fallowfield et al. demonstrated that MMP-13 was mainly expressed by scar-associated macrophages in liver fibrosis, and its gene deletion resulted in a retarded resolution of fibrosis [151]. Furthermore, MMP-2 has been reported to exert a protective effect against the progression of fibrosis in liver by inhibiting type I collagen synthesis [152] or by suppressing TIMP-1 upregulation instead of its direct proteolytic action [153]. In contrast, however, it has been reported that MMP-13 mediates the initial inflammation and thereby contributes to the acceleration of fibrogenesis in cholestatic livers [154]. Furthermore, the positive involvement of MMP-19 in the development of liver fibrosis was indicated by a study showing that MMP-19-deficient mice showed impaired signaling of transforming growth factor-beta (TGF-β), a major pro-fibrotic cytokine, and attenuated liver fibrosis in the disease model mice [155]. Although ADAM-28 was also upregulated in the liver tissue of patients with chronic liver diseases and its expression levels correlate with the histological degrees of fibrosis, the etiological relationship between this enzyme and fibrosis remains unclear [156].

#### 4.4.2. Inflammatory Bowel Diseases

IBD, such as ulcerative colitis and Crohn’s disease, are chronic diseases associated with inflammation and remodeling of gastrointestinal tract tissues. In IBD, a dysregulated response of the intestinal immune system toward intraluminal flora in genetically-predisposed patients causes the activation and release of several inflammatory mediators, including cytokines, nitric oxide, eicosanoids and proteolytic enzymes, which in turn trigger a cascade of events resulting in the intestinal injury [157].

MMPs play important roles in the pathophysiology of IBD, where they have been shown to regulate epithelial barrier function, immune response, angiogenesis, fibrosis and wound healing beyond simple ECM degradation [157,158]. In experimental colitis of animal or human gut explant models, MMP-3, -7, -9, -12 and TIMP-1 expression was elevated, and several MMP inhibitors significantly reduced tissue injury and inflammation [159,160]. Pender et al. demonstrated a direct effect of recombinant MMP-3 to produce rapid severe tissue injury in a human fetal gut explant model, indicating the functional involvement of MMP-3 in IBD [161]. Double knockout mice lacking both gelatinases, MMP-2 and -9, were resistant to the development of experimental colitis [162]. Further, an important role of MMP-12 in IBD pathogenesis was suggested by a study showing that MMP-12 knockout mice were protected against drug-induced colitis [163]. Kobayashi et al. identified MMP-3 and -10 belonging to the same subgroup of stromelysins as the major contributors to drug-induced colitis, on the basis of their inhibition by siRNA or blocking of the signaling pathways, resulting in effectively reduced severity of colitis [164]. Similarly in human, the expression and proteolytic activity of MMPs, including MMP-1, -2, -3, -7, -9, -10, -12 and -13, have been demonstrated to be elevated in inflamed IBD mucosa or serum of IBD patients [165,166,167,168], and some of these levels corresponded to the severity of the disease or inflammation [169,170,171]. In addition, several functional polymorphisms in the promoter region of the *MMP-3* gene were associated with increased susceptibility to IBD [172,173]. Recently, a wide range of cellular sources of MMPs has become increasingly evident, such as epithelial cells, mesenchymal cells and leukocytes [174]. MMP-9 is one of the most abundantly-expressed proteinases in IBD, which was markedly upregulated in small mononuclear leucocytes, granulocytes and giant cells in intestinal fistulae of IBD patients, suggesting its contribution to fistula formation [175]. Lakatos et al. reported that the mucosal upregulation of MMP-9 correlated with the severity of inflammation in IBD [169]. Based on these findings, selective MMP-9 inhibition has currently been regarded as a promising therapeutic strategy for the treatment of IBD [176].

### 4.5. Renal Disorders

A key pathological characteristic in renal disorders is alterations in the ECM components of kidney, leading to parenchymal destruction. Accumulating evidence has implicated dysregulation of MMPs in a wide diversity of renal disorders involving their pathological processes. Although the temporal and spatial expression of MMPs in the kidney is too complex to be fully elucidated, MMP-2, -3, -9, -13, -14, -24, -25, -27, -28 and TIMP-1, -2, -3 have been demonstrated to be expressed in various sites of the organ [177]. 

#### 4.5.1. Acute Kidney Injury

In various models of acute kidney injury (AKI), which are mostly produced by renal ischemia-reperfusion injury, not only increased expressions of MMP-2, -9 and TIMP-2, but also decreased expression of TIMP-1 have been observed [178,179]. The increased MMP-9 activity during ischemic injury of rat kidneys was associated with degradation of tight junction proteins in both the endothelial cell fraction and glomeruli, leading to increased vascular permeability, which is consistent with characteristic of AKI [178,179]. A recent in vitro study with human proximal tubular cells demonstrated that MMP-3 was upregulated by physiological stimuli during AKI and induced kidney injury molecule-1, known as an emerging therapeutic target and a biomarker for kidney injury [180]. Decreased MMP activity by pharmacological inhibitors or genetic deletion generally resulted in amelioration of renal injury in experimental models of AKI [181,182,183,184,185], some of which were associated with decreased oxidative stress [183,184] or microvascular stability, partly by preserving the tissue level of vascular endothelial growth factor [185]. These data support the functional contribution of MMPs to the pathological mechanisms underlining AKI. In contrast, Bengatta et al. reported that MMP-9 could play a protective role against AKI-associated apoptosis in the S3 segment of proximal tubule and the intercalated cells of collecting duct, most likely by releasing a soluble form of stem cell factor [186]. On the other hand, the role of ADAM/ADAMTS in AKI has not yet been clarified. More recently, however, ADAM-10 has been demonstrated to be the major sheddase responsible for release of meprin A during AKI [187]. Meprin A is a membrane-associated metalloproteinase in proximal tubule and known to play an important role in AKI.

#### 4.5.2. Chronic Kidney Disease

CKD is a major public health concern, and its final common feature is kidney fibrosis, like glomerulosclerosis and tubulointerstitial fibrosis, resulting from chronic injury and inflammation [188]. Kidney fibrosis is characterized by substantial accumulation and activation of interstitial myofibroblasts responsible for the excessive deposition of ECM [189], and a process described as epithelial-to-mesenchymal transition (EMT) is proposed as a major source of the myofibroblasts [190].

In several animal models of renal fibrosis, early increases in MMP-2, TIMP-1 and TIMP-3 expression and activity have been observed, suggesting accelerated ECM turnover following injury [177]. At earlier periods, MMP-2 structurally alters the tubular basement membrane, which triggers tubular EMT with resultant tubular atrophy, fibrosis and renal failure [191]. MMP-2 deletion and minocycline treatment in mouse obstructive nephropathy showed amelioration of renal fibrosis via inhibition of EMT and macrophage infiltration [192]. In patients with CKD, elevated serum and plasma levels of MMP-2 and MMP-9 have been found [193,194]. Tan et al. demonstrated in an in vitro study that MMP-9, as well as MMP-2 derived from macrophages could directly induce the tubular cell EMT to an extent similar to TGF-β [195]. This finding is supported by an in vivo study where mice lacking the *MMP-9* gene blocked tubular EMT, resulting in a reduction of interstitial fibrosis in obstructive nephropathy [196]. More recently, MMP-7 was also reported to induce EMT [197]. On the other hand, increased TIMP-1 and -2 expression in glomeruli was found in patients with glomerulosclerosis [198], and elevated urine concentrations of TIMP-1 in CKD patients correlated with those of tenascin, an ECM glycoprotein [199]. These findings might suggest the reduced ECM degradation favoring the development of renal fibrosis at later time points.

#### 4.5.3. Diabetic Nephropathy

Diabetic nephropathy is the most common cause of end-stage kidney disease, which is clinically characterized by albuminuria and progressive renal failure. It is pathologically characterized by thickened basement membrane and expansion of the glomerular mesangial matrix and tubulointerstitial space, which is due to excessive deposition of ECM [200]. Multiple studies have demonstrated the upregulated levels of MMP-2, -7, -8 and -9 in serum and urine from patients with type 1 or type 2 diabetes [201]. In addition, the urinary MMP-9 concentration was reported to be correlated with the degree of albuminuria in type 2 diabetic nephropathy [202]. At the moment, however, the role of these MMPs in the pathogenesis of diabetic nephropathy remains controversial. Concretely, some models showed that decreased MMP activity, such as MMP-2 and -9, could ameliorate the kidney lesion [203,204], whereas in other models, local delivery of the *MMP* gene prevented the onset of diabetic nephropathy [205]. These contradictory findings might highlight the complexity of MMP pathobiology in this disease.

### 4.6. Respiratory Disorders

Normal lung function requires alveolar support by ECM. Therefore, abnormal remodeling or excess destruction of ECM could cause many pulmonary disorders, including chronic obstructive pulmonary diseases (COPD), interstitial lung disease, bronchial asthma and tuberculosis. The lung ECM mainly consists of type I collagen and elastin, and their degradation is known to be accomplished by MMPs and other proteinases.

#### 4.6.1. Pulmonary Emphysema

Pulmonary emphysema is a major component of the morbidity and mortality of COPD, in which cigarette smoke is the most common etiologic agent. Enlargement of the peripheral airspaces of the lung, resulting from destruction of alveolar wall matrix structures and leading to airflow obstruction, is a hallmark of this disease [206]. Alveolar macrophages are a major source of MMPs, including MMP-1, -9 and -12, which have been implicated in emphysema formation [207,208].

D’Armiento et al. showed that MMP-1 overexpression in lungs of transgenic mice caused pulmonary emphysema, proposing the proteinase/anti-proteinase imbalance theory in the pathogenesis of emphysema [209]. Indeed, MMP-1 expression was upregulated in bronchoalveolar lavage fluid from patients with emphysema [210]. It was also demonstrated that cigarette smoke directly induced MMP-1 mRNA and protein expression and then increased the collagenolytic activity in human airway epithelial cells [211]. The contribution of MMP-9 to pulmonary emphysema development was indicated by a study of transgenic mice showing that overexpression of this proteinase in macrophages could alter the ECM, leading to the progressive enlargement of air space in lung, which is a similar finding to human emphysema [212]. MMP-12 has been most extensively investigated and shown to play a consistent critical role in animal models of emphysema. Exposure to cigarette smoke consistently upregulated MMP-12 expression in various experimental models [213,214]. In addition, mice lacking MMP-12 were completely protected from the development of emphysema induced by cigarette smoke [215]. Treatment of guinea pigs with MMP-9/MMP-12 inhibitor for six months of smoke exposure was also protective, which was accompanied by decreases in lavage neutrophils and macrophages [216]. In contrast, existing human data with some contradictions in this regard are hard to interpret and remain controversial [217]. However, a relatively recent study in patients identified a polymorphism in MMP-12 (rs2276109) as a positive effecter on lung function in smoking adults, and that was related to a reduced risk of COPD [218]. On the other hand, the polymorphism of the *ADAM-33* gene was reported to be a relevant risk for COPD, but there is little evidence for a relationship between ADAMs/ADAMTSs and emphysema [219].

#### 4.6.2. Interstitial Pulmonary Fibrosis

Pulmonary fibrosis is a severe and refractory disease with an extremely poor prognosis.

Its histological feature is the replacement of normal alveolar architecture with collagen-rick fibrotic matrix [220]. Fibrosis can result from the aberrant remodeling during recovery from lung injury, when there is a disequilibrium process between the synthesis and degradation of ECM components [221].

Several metalloproteinases, including MMP-1, -2, -3, -7, -9 and -14, are highly expressed in the lung of patients with pulmonary fibrosis [222]. Among them, MMP-7 was the most significantly increased gene in an oligonucleotide microarray analysis [188]. Despite the proteolytic activity of MMPs, most experiments with MMP knockout mice have revealed protection from bleomycin-induced lung fibrosis, rather than increased collagen accumulation [188,223,224,225]. The profibrotic property of these enzymes can be explained by the fact that they mediate the cleavage of basement membrane collagen during the acute phase of lung injury, allowing fibroblasts to migrate into the alveolar compartment and subsequently produce collagen, leading to fibrosis [225,226]. Moreover, it was demonstrated that ADAM-19 and ADAMTS-9 were upregulated and also contributed to the collagen production in lung cells exposed to TGF-β [227].

#### 4.6.3. Asthma

Asthma is a common chronic inflammatory disease of the airway, which is pathologically characterized by an increase in epithelial mucin store caused by surface epithelial mucus metaplasia and by increased numbers of subepithelial bronchial microvessels that become leaky during inflammation [228].

Patients with asthma showed an increased gelatinolytic activity derived from MMP-2 and MMP-9 in their sputum [229]. Both MMP-2 and MMP-9 could promote the egress of inflammatory cells into airway lumen, an essential mechanism by which each MMP could exhibit a protective anti-inflammatory role in asthma. In accordance with this notion, lack of MMP-2 or MMP-9 in animal models resulted in heightened airway inflammation and increased susceptibility to asphyxiation induced by allergens [230,231]. In addition, a study with double knockout mice lacking MMP-2 and MMP-9 revealed a dominant role of MMP-9 in the resolution of allergic inflammation overlapping with a more limited role of MMP-2 [232]. MMP-8 has also been demonstrated to play a protective role in asthma by promoting the clearance of recruited neutrophils [233]. In contrast, a common serine variant of the *MMP-12* gene in human (rs652438) resulting in more aggressive ECM degradation was positively associated with the severity of asthma [234]. This was confirmed by pharmacologic inhibition of MMP-12 in allergen-sensitized sheep that alleviated both early and late airway responses to allergen challenge [234,235]. Similarly, MMP-12 deficiency in the allergen-induced mice showed a significant improvement in allergic airway inflammation induced by cockroach antigen [236]. There are some other MMPs that have been implicated in the pathology of asthma, such as MMP-1, -3, -7 and -19 [228], as well as ADAM-33 [237].

#### 4.6.4. Tuberculosis

Tuberculosis (Tb) is still a global health pandemic, where *Mycobacterium tuberculosis* needs the destruction of the lung ECM to spread from interstitium into the airway and to cause cavity formation, an immunodeficient site where *Mycobacterium tuberculosis* can proliferate [238]. The underlying mechanism of matrix destruction in Tb remains to be fully elucidated, but it must involve the action of proteinases, particularly MMPs, which are able to degrade the structural fibril of lung [239].

The first description of increased MMP expression in human Tb was in a small study showing elevated MMP-9 levels in bronchoalveolar lavage fluid from two patients with Tb [240]. Subsequently, a number of studies focusing on specific MMPs have been reported. For example, MMP-1, -2, -8 and -9 were found to be upregulated in the pleural fluid of patients with Tb compared to heart failure patients [241]. Furthermore, circulating levels of MMP-9 were reported to be correlated with the severity of Tb [242]. Despite the frequency of investigations on MMP-9 in Tb, probably due to its relative abundance and ease of detection by gelatin zymography, it cannot degrade fibrillar collagen and, therefore, may not be the final effector of matrix destruction in pulmonary Tb [239]. Elkington et al. demonstrated that *Mycobacterium* *tuberculosis* specifically increased *MMP-1* gene expression and secretion in primary human macrophages [243]. Consistent with this, a microarray analysis with human macrophages revealed that MMP-1 was the most highly upregulated gene, being 256-fold higher in patients with pulmonary Tb compared to controls [244]. Moreover, the functional importance of MMP-1 in Tb-related tissue destruction was suggested by a study with human MMP-1 transgenic mice showing increased collagen destruction in Tb granulomas [245]. Taken together with these findings, this enzyme is likely to be the primary MMP and the most responsible for the destruction of ECM leading to disease development in pulmonary Tb [239].

## 5. Therapeutic Implications

The MMP family has still been gathering more and more considerable attention as essential proteinases involved in various pathological events, as mentioned in this review. Accordingly, the accumulated evidence of the pathophysiological roles of MMPs in many diseases has made MMPs promising therapeutic targets. Until now, a great number of synthetic MMP inhibitors has been developed and showed certain effects, at least in experimental models [246]. However, all of the results from previous clinical trials using MMP inhibitors for the treatment of cancer and non-neoplastic disorders, including arthritis and vascular diseases, disappointingly failed [60,247,248]. There are some reasons for these failures; problems of the clinical trial itself (such as poor trial design and inadequate clinical end points) and the drug themselves (such as metabolic instability, low oral bioavailability, poor inhibitory specificity and adverse side effects) [6]. Non-selective broad MMP inhibitors in early clinical trials caused unwanted side effects, including musculoskeletal pain probably due to the inhibition of MMP-1 or ADAM-17 [249], indicating that not all MMPs need to be blocked in all tissues and at all times [250]. Therefore, for instance, developing a site-specific delivery system or MMP inhibitors with higher selectivity may be helpful. Recently, structured-based design techniques have contributed to the search for selective MMP inhibitors, and these agents can also be derived from natural products [251]. There are now some selective MMP inhibitors developed for the treatment of the non-neoplastic disorders and cancer, such as MMP-13 inhibitors in OA [252], MMP-14 inhibitors in RA and cancer [253,254] and MMP-12 inhibitors in COPD [255], all of which have been proven to be effective in animal models.

Most notably, the actual roles of MMPs, ADAMs and ADAMTSs in diseases have been increasingly proven more complex than expected. One MMP, in fact, can have different or rather opposing effects based on the cell or tissue types along with their conditions [256]. Furthermore, the broad substrate spectrum, functionally overlapped or interacted proteolytic effects, and the4 wide distribution of MMPs make it extremely hard to choose which MMPs to target for therapeutic purposes. To fully understand the roles of MMPs in health and disease, current research works have employed new modalities, such as in vivo molecular imaging probes or more specific antibodies for individual MMPs [257,258]. These tools can detect the localization of specific MMP activity and, thus, aid in the development of MMP inhibitors by determining effective and safe dose ranging and providing therapeutic efficacy in vivo.

Even now, novel regulatory or functional mechanisms of MMP action are being discovered. For example, it was recently reported that several MMPs, such as MMP-1, -2, -3, -11 and -12, can act also on intracellular proteins [259,260,261,262,263]. While further detailed information about this action will be required, the intracellular MMP activity of a specific cell type may represent a new therapeutic target. Moreover, upstream factors of MMPs regulation, such as the Toll-like receptor 4/NF-κB signaling pathway, serve as other potential targets of inhibition [264]. On the other hand, there is evidence of an improvement in the MMP/TIMP ratio with diet and exercise [265,266].

## 6. Conclusions

In the current review, we widely discuss the recent advances made in understanding the role of MMPs, especially in the context of non-neoplastic disorders. Despite considerable research efforts over the past 50 decades, many emerging findings related to MMPs are still being reported from research laboratories around the world every year, and that makes the role of MMPs in vivo more and more complex and important. There are fundamental challenges to be overcome in applying the MMPs-targeted therapy in the clinical setting. Nonetheless, it is hoped that the previous, ongoing and future studies will together translate their findings into novel medical strategies for cancer and non-neoplastic disorders soon.
